# Showup identification decisions for multiple perpetrator crimes: Testing for sequential dependencies

**DOI:** 10.1371/journal.pone.0208403

**Published:** 2018-12-06

**Authors:** Nina Tupper, Melanie Sauerland, James D. Sauer, Nick J. Broers, Steve D. Charman, Lorraine Hope

**Affiliations:** 1 Maastricht University, Department of Clinical Psychological Science, Maastricht, The Netherlands; 2 University of Portsmouth, Department of Psychology, Portsmouth, United Kingdom; 3 University of Tasmania, Division of Psychology, Tasmania, Australia; 4 Maastricht University, Department of Methodology and Statistics, Maastricht, The Netherlands; 5 Florida International University, Department of Psychology, Miami, Florida, United States of America; Psychologische Hochschule Berlin, GERMANY

## Abstract

Research in perception and recognition demonstrates that a current decision (*i*) can be influenced by previous ones (*i–j*), meaning that subsequent responses are not always independent. Experiments 1 and 2 tested whether initial showup identification decisions impact choosing behavior for subsequent showup identification responses. Participants watched a mock crime film involving three perpetrators and later made three showup identification decisions, one showup for each perpetrator. Across both experiments, evidence for sequential dependencies for choosing behavior was not consistently predictable. In Experiment 1, responses on the third, target-present showup assimilated towards previous choosing. In Experiment 2, responses on the second showup contrasted previous choosing regardless of target-presence. Experiment 3 examined whether differences in number of test trials in the eyewitness (vs. basic recognition) paradigm could account for the absence of hypothesized ability to predict patterns of sequential dependencies in Experiments 1 and 2. Sequential dependencies were detected in recognition decisions over many trials, including recognition for faces: the probability of a *yes* response on the current trial increased if the previous response was also *yes* (vs. *no*). However, choosing behavior on previous trials did not predict individual recognition decisions on the current trial. Thus, while sequential dependencies did arise to some extent, results suggest that the integrity of identification and recognition decisions are not likely to be impacted by making multiple decisions in a row.

## Introduction

In October 2015, news outlets [1] featured security footage of an unresolved case: the attempted abduction of a truck driver on the French-Belgian border. As the truck driver walked around the rear of his truck, two men appeared and attacked him. While the two perpetrators struggled to force the driver into the back of a waiting car, an elderly passerby intervened, pulling at the perpetrators’ jackets and trying to place himself between them and the truck driver. Following the failed abduction and a hurried, but equally unfruitful search for the truck driver’s keys, the two men fled the scene by car.

This case is just one example of the many violent crimes that are committed by multiple perpetrators. Gang violence [2], hate crimes [3], rapes [4] and assaults [5] are often committed by perpetrators working together. In fact, the rising rate of such crimes appears to be a global phenomenon. For example, in Finland, Sweden, and the Netherlands, 13–17% of homicides between 2003 and 2006 involved two or more perpetrators [6] while the proportion of homicides with multiple perpetrators in the U.S. reached 20% in 2008 (nearly double that reported in 1980 [7]). These crimes often involve victims or bystanders as eyewitnesses—like the driver and the passerby above—who may be asked to identify multiple suspects related to the multiple perpetrators. Yet, the decades of research focused on uncovering and understanding factors that affect accuracy in eyewitness identification procedures typically considers only identifications of a single perpetrator, providing little empirical evidence to support or oppose recommendations in protocols specific to the context of multiple perpetrator crime. Should police departments, for instance, follow the example of the U.K. and multiply “best practice” by creating a new lineup for each suspect of a different perpetrator [8]? If so, does the order of presentation of identification tests affect the reliability of the evidence obtained? Or does the act of making multiple identification decisions affect the decisions themselves?

In this paper, we address this last question, examining the consequences of testing memory for multiple perpetrators [9]. We present three experiments examining whether current showup identification decisions are associated with witness choosing behavior on previous showup decisions. We aimed to determine whether sequential dependencies (i.e., whether choosing behavior on previous tests influences choosing on a current test) should be considered in cases when eyewitnesses are asked to make multiple identification decisions, specifically when those decisions pertain to the different suspects in a multiple perpetrator crime.

### Identification of multiple perpetrators

Clifford and Hollin [10] first revealed the difficulty of eyewitness identification in the context of multiple perpetrator crimes when they had participants view a non-violent event with one, three, or five perpetrators. Despite only having to select the main perpetrator from a target-present lineup immediately following the crime, only 30% of participants in the three-perpetrator condition and 20% in the five-perpetrator condition made accurate identifications (compared with 40% in the one-perpetrator condition). More recently, Megreya and Bindemann [11] demonstrated a similar drop in accuracy with as few as two unfamiliar faces to be encoded. Participants viewed a mock crime with one perpetrator alone or with an accomplice and were subsequently asked to identify the perpetrator. The presence of a second person at encoding was associated with decreased identification accuracy in target-present lineups (lower hit rates and higher miss rates). Approximately 54% of participants who saw the perpetrator alone were able to accurately identify him/her, compared with only 29% of participants who saw the perpetrator with an accomplice.

To date, three procedures have been proposed to address the applied issue of the multiple perpetrator identification disadvantage. The *two-person serial lineup* [12], the *elimination lineup* [13, 14], and an adapted *sequential identification procedure* [15] were each tested against traditional simultaneous lineups, sequential lineups, or both. The results were mixed, and any improvements associated with these methods depended upon which target identity was being presented (i.e., accomplice vs. perpetrator), the presence or absence of the target in the lineups, or both. Unfortunately, when these new methods fall short, we do not know if it is because the proposed adaptations did not address the mechanisms they intended to, or if the theories used to justify these adaptations are ultimately not relevant to the multiple perpetrator identification disadvantage. For example, the two-person serial lineup is intended to provide context to aid memory by presenting the sequential lineups of the culprit and of the accomplice at the same time [12]. Although the lineups for each are flashed side-by-side on the screen, the two suspects are never shown simultaneously, but always paired with a filler. In theory, the context of one face should aid our ability to recognize or reject the other face. But when this lineup does not improve identification accuracy, is it because contextual cuing is not useful for faces in a lineup context? Or is it because the suspects are never shown together, and thus are not cuing memory? Perhaps it is difficult to interpret their results because they are premature attempts to fix problems that are still not well understood, meaning the adapted lineups amount to trial-and-error solutions.

Shallow encoding [16] and increased memorial demand [17] have more recently been explored as reasons for the decreased identification performance for multiple perpetrator crimes, and both appear to play a role. However, there is another independent factor that is unique to multiple perpetrator identification that has yet to be considered: the decisional structure of making multiple identifications. Below, we explore how the act of making multiple identifications may undermine the integrity of those decisions.

### Sequential dependencies in perception and recognition

An individual police lineup has been likened by researchers to a real-world signal detection decision, but with the modification to include filler (i.e., non-suspect) misidentifications [18, 19]. The signal detection model, however, mathematically assumes independence of trials, for which a decision is based solely on the evidence present in that trial. In contrast, research in perception and memory demonstrates that a current decision (*i*) can be influenced by a previous one (*i–j*), so that a current response may favor (assimilation) or disfavor (contrast) the preceding responses [20]. In other words, in a series of trials presented one-after-another, the responses, although separate, are not independent. These *sequential dependencies* appear in perception, classification, and recognition tasks where participants make multiple, sequential decisions—tasks that present a theoretical overlap with making multiple eyewitness identification decisions.

Sequential dependency can be demonstrated in its simplest form in a traditional detection experiment. Howarth and Bulmer [21] seated participants in a dark room with a flash-bulb set at a 50% detection rate at a given intensity, meaning that the light was bright enough to be detected, but dim enough that participants only reported seeing it half of the time. The momentary flashes were accompanied by the sound of a bell, so that when participants heard the bell ring, they indicated whether or not they had seen the flash of light (*yes* vs. *no*). At 50% detection, participants will make errors half of the time; errors that should theoretically display natural fluctuations and therefore appear randomly throughout the hundreds of trials. However, participants demonstrated a tendency to assimilate responses towards previous ones, meaning that a *no* response was more likely to be followed by another *no* response than a *yes* response. Further still, at some points, the light signal was omitted so that the bell rang without the accompanying light flash. When the experimenters forced a sequence of three of these blank trials (*no-no-no*), they found the same degree of assimilation for the subsequent fourth response as for three natural occurring negative responses. Such sequential dependencies are found in a variety of tasks, including absolute judgments of sound [22] and the perceptual classification of facial expressions [23].

The mechanism underlying sequential dependencies remains a subject of debate, with attempts to model sequential dependencies favoring one of the two systems involved in a perception task: decisional processes and the cognitive system. Some models consider sequential dependencies to arise from biased decision-making [20]. According to these models, assimilation results from the observer’s short-term assumption that the most recent stimulus is also the most likely to occur again. However, patterns of contrasting answers are the result of the observer attempting to correct decisional criteria to a desirable level in the long-term. These fluctuations in response bias purport to explain why judgments show assimilation immediately following trial *i*, but revert to contrast after a few trials. On the other side of the debate are models arguing that sequential dependencies arise either entirely, or at least in part, from the cognitive system [24, 25, 26]. In these models, sequential dependencies arise as a result of inappropriate information being carried forward from the previous trial, affecting the perception of the current stimulus.

Malmberg and Annis [27] were the first to demonstrate sequential dependencies in recognition memory. They presented a series of experiments using traditional recognition paradigms and judgments of frequency recognition tasks to approximate the perception and categorization tasks that routinely demonstrate sequential dependencies. For example, in one experiment, participants studied 40 word pairs and were later tested on their recognition for those words among never-studied words. As with Howarth and Bulmer’s [21] light-detection experiment, participants were more likely to respond *old* if they had responded *old* (rather than *new*) on the previous trial, regardless of whether the previous response was correct (hit) or incorrect (false alarm). The appearance of sequential dependencies was consistent across several replications with different stimuli, including landscape images, and picture-word pairs.

### The current research

Studies investigating the cause of the multi-face recognition disadvantage [16, 17] tend to focus on the encoding conditions: how factors that affect perception and attention interfere with encoding, and thus damage chances of identification from the outset. Consequently, studies adapting lineups that were originally designed for single-perpetrator crimes so far considered these encoding difficulties and adjusted methodology in attempts to compensate for the resulting impoverished memory [12, 15]. While this is a reasonable starting point to investigate multiple perpetrator identifications, it is also important to explore other factors that may affect identification decisions. In this vein, we investigated the possibility of sequential dependencies within the eyewitness paradigm. Specifically, how does the act of making multiple identification decisions for unique perpetrators affect the validity of those decisions? Some research has considered the impact of making multiple showup identification decisions for a single-perpetrator crime [28, 29], the non-independence of multiple identification decision remains untested in the context of eyewitness identification for multiple suspects related to the multiple perpetrators of the same crime.

Multiple perpetrator crimes present a framework in which relatively few sequential decisions are made, and in which these decisions have serious consequences. Sequential dependencies measured in the recognition paradigm have little substantial effect on overall recognition accuracy because the beneficial and detrimental sequences of dependencies will typically balance out over the many trials, reducing its impact on the overall accuracy for recognition [27]. Considering identification paradigms lack the many trials needed to balance out recognition accuracy, the appearance of sequential dependencies in this context would be a matter of substantial impact and cause for concern. Therefore, we tested for sequential dependency effects within the eyewitness identification context by having participants make multiple, consecutive showup decisions.

Show ups were chosen because they are particularly well-suited for an initial test for sequential dependencies within the eyewitness context for three reasons. First, although lineups are advisable because they reduce the probability of misidentification by random chance, showups (live or photographic) are still a common identification procedure around the world [28, 30, 31]. Second, forced-report showup decisions (*Is this the perpetrator*? *Yes vs*. *no)* emulate the binary-decision tasks in which sequential dependencies have been consistently observed. Third, showups permit a controlled investigation of sequential dependencies on identification decision-making free from the influence of lineup construction variables (e.g., filler similarity, lineup presentation method) or from the statistical noise of making comparisons between fillers. If sequential dependencies are found to affect showup decision-making, subsequent investigations can determine how these effects interact with lineup composition and presentation variables.

Across two initial experiments, we examined the relation of previous identification decisions to subsequent choosing behavior in the context of the multiple showup identification decisions for a multiple perpetrator crime. If it is possible to predict current choosing on a showup identification decision from previous choosing, it provides initial evidence that sequential effects may be present in multiple showup identification decisions. Given that research has previously demonstrated that sequential dependencies in recognition are a result of interference from previous trials [27], Experiments 1 and 2 consider both previous signal (target-presence: present vs. absent) and previous response (Choosing: *yes* vs. *no*) as predictors of current choosing behavior [25]. We expected that initial showup responses would predict choosing for subsequent showup responses. In other words, choosing on a previous showup identification would be associated with choosing on subsequent ones, and rejecting on a previous showup identification would be associated with rejecting on subsequent ones. We also expected previous target-presence to be separately associated with the current identification decision, such that the previous target being present would predict current choosing and the previous target being absent would predict current rejecting [25].

We also considered the possibility of an interaction between current target-presence and previous choosing on current choosing, such that being confronted with a target-absent trial would further raise the probability of rejection given a previous rejection. Non-memorial factors tend to exert stronger effects on recognition memory tasks when the target stimulus is absent and there is no opportunity for genuine recognition [32]. In other words, if memory is not able to provide the answer, people look for other cues to influence their decision. In this way, sequential dependencies might represent an attempt to use imperfect cues to guide decision-making under conditions of uncertainty [33]. Straight forward sequential dependencies should arise regardless of target presence, but it is possible that the strength of the effect will vary depending on whether the target is present or not.

Although Experiments 1 and 2 were conducted separately, they used similar methodologies and analyses to answer the same question. Thus, although the data are not collapsed across experiments, the methods and results are presented together.

## Experiments 1 and 2

### Ethics statement

These studies were approved by the ethical review board of the Faculty of Psychology and Neuroscience of Maastricht University. Written consent was obtained from participants in Experiment 1. Participants in Experiment 2 provided consent by clicking the button to continue the experiment.

### Participants and design

A total of 411 participants were tested, 404 of which were included in analyses. Participants either completed the experiment in the lab (Experiment 1, *N* = 120) or online (Experiment 2, *N* = 291). The average age of participants was 21 years (*M* = 20.77, *SD* = 3.64). They were compensated with a €5 gift voucher (Experiment 1) or participation credit (Experiments 1 and 2).

Participants viewed a three-person mock crime video and were subsequently presented with three photographic showups, one for each of the three perpetrators. In Experiment 1, we aimed to provide an initial test of sequential dependency in facial identification. Four conditions were chosen to optimize conditions for sequential dependencies through an established pattern of target-present and target-absent showup photographs [21]. The first and second showups were always consistent in target-presence; they were either both target-absent (TA) or both target-present (TP), while the third showup was either consistent or different, leading to four conditions with targets: (1) TA/TA/TP, (2) TA/TA/TA, (3) TP/TP/TA, and (4) TP/TP/TP. In retrospect, we realized this also meant that we were not able to disentangle the effect of target-presence between showups 1 and 2 on showup 2. Thus, Experiment 2 implemented all combinations of target presence by adding four additional conditions with targets: (5) TA/TA/TP, (6) TA/TP/TA, (7) TP/TA/TP, and (8) TP/TA/TA. Presentation order of targets (i.e., 123, 132, 231, 213, 312, 321) was counterbalanced for both experiments.

### General method for Experiments 1 and 2

#### Materials

**Crime video.** In the 2:45 min mock crime video, the male victim arrives by bike and locks it against a railing with other bikes. Three target people, one woman and two men, are shown in the background gesturing towards the victim. When the victim walks into a nearby building, the thieves use a hand-saw to break the locks of two bikes, including the victim’s, and walk away with the bikes. Each target actor in the video has approximately 15–20 s of close-up shots in which their faces are clearly visible.

**Showups.** Three target-absent and three target-present showups were constructed, one for each of the three perpetrators. The showups consisted of color photographs 4.39 x 5.89 cm in size. The targets were photographed on the same day as the stimulus event was filmed, but wore different clothing. One innocent suspect was selected as a replacement for each target in the target-absent showups. The replacements were chosen based on similarity to the actual target, as established by a pilot study with *N* = 22 participants (age: *M* = 27.45, *SD* = 12.14). Specifically, replacements were rated as statistically similar to the perpetrator with regard to memorability, distinctiveness, and typicality [34; 35]. Participants were also asked to judge the similarity of the target faces paired with each of their possible replacements. This comparison score was used to match for similarity across the three target-replacement pairings, so that each of the three target-absent showups would be equally difficult for participants to judge. Results of the pilot study are available in supplementary materials (S1 Table).

#### Procedure

Participants arrived at the lab for individual testing sessions (Experiment 1) or received a Qualtrics [36] link to complete the experiment online (Experiment 2). Participants were informed that the experiment would be administered using a self-paced computer task. After giving informed consent, participants were told that they would be shown a video and were instructed to pay close attention as they would be asked questions about it later. After watching the mock crime video, participants completed a 20–30 min filler task by answering a series of questionnaires (Experiment 1 and 2) or by completing a combination of search tasks and word-generation games (Experiment 2). Next, participants were reminded that they had seen a film of three thieves stealing a bike, and were now considered eyewitnesses. They were instructed: *You will be shown a series of three photographs*. *Each photograph is one suspect for each of the three bike thieves*. *For each photograph*, *please decide whether or not the person shown was one of the perpetrators*. *Once you make a decision*, *you will move on to the next photo*. A subsequent screen displayed a one-time warning that the persons in the photographs may or may not be the actual perpetrators.

Participants were then shown a photo for one of the perpetrators (Suspect 1: Present or absent). A forced-report question asked if the person shown was one of the perpetrators (*yes* or *no*), after which they were asked to indicate how certain they were in their decision (0–100%). The procedure was repeated for Suspects 2 and 3. Although suspects are numbered here for convenience, presentation order of targets was counterbalanced; meaning Suspect 1 for the eyewitness could correspond to any of the three perpetrators. Following all identification decisions and confidence ratings, participants were shown the photos of those they had positively identified and asked to name the role each played in the crime. However, role assignment and confidence are outside of the scope of the current research and are therefore not addressed further. Finally, participants were thanked for their time and debriefed.

Experiment 2 differs from Experiment 1 in two ways. In order to determine whether participants had watched the entire video, a still image of a white arrow and the text “*This is a white arrow*, *please remember this arrow as you will be asked about it later*” was added for the last 7 s of the video (after the target event). Following the filler task, participants were asked to name the shape and color presented at the end of the video. This section of the computer task was timed so that the task advanced automatically after 2:52 min regardless of whether or not the video was paused. Therefore, participants who could not correctly name the shape and color (*n* = 4) were assumed to have not completed the video and were removed from all analyses. A final question prompted participants to describe the environment in which they completed the experiment (e.g., time of day, location, presence of others).

### Results

In Experiment 1, all 120 participants were retained for data analysis. In Experiment 2, seven participants were removed from data analysis for answering the control questions incorrectly (4), not completing the filler task (2), or because Qualtrics recorded their experiment duration time as exceeding four hours and the participant did not respond to requests to elaborate (1), leaving 284 participants.

#### Descriptive statistics for choosing on showups

Across the three showup identification decisions in both Experiments 1 and 2, choosing rates were low, at 34–42%. Overall, only 4–12% of participants chose on all three showups. Meanwhile, 15–22% of participants rejected all decisions. Less than half of participants (26–47%) chose on at least two showups. See Table 1 for choosing rates for each experiment.

**Table 1:** Experiments 1 and 2: Proportion (frequency) of choosing across showups and overall.

	Choosing by showup			Overall Choosing			
	Showup 1	Showup 2	Showup 3	0 chosen	1 chosen	2 chosen	3 chosen
**Expt. 1**				.15 (9)	.38 (23)	.35 (21)	.12 (7)
TP	.53 (32)	.53 (32)	.52 (31)				
TA	.25 (15)	.23 (14)	.27 (16)				
Overall	.39 (47)	.38 (46)	.39 (47)				
**Expt. 2**				.22 (62)	.43 (120)	.30 (84)	.04 (12)
TP	.54 (76)	.43 (62)	.58 (82)				
TA	.25 (36)	.25 (35)	.29 (39)				
Overall	.41 (114)	.34 (93)	.42 (117)				

Displayed under “Choosing by showup” are proportions of participants choosing on target-present and target-absent showups. Displayed under “Overall Choosing” are proportions of participants who chose on zero, one, two, or three showups. Raw frequencies are between parentheses. TA denotes target-absent showups and TP denotes target-present showups.

#### Experiment 1: Testing for sequential dependencies

In order to establish the association of previous identification decisions and both previous and current target-presence with current identification decisions, we performed separate binary logistic regressions for choosing on the second and third showup. For example, for choosing on the second showup, we entered previous target-presence (absent vs. present on Showup 1), current target-presence (absent vs. present on Showup 2) and previous choosing (*yes* vs. *no* on Showup 1) as predictors. For choosing on the third showup, we used previous target-presence, current target-presence (Showup 3), and previous choosing (*yes* vs. *no* on Showup 1 and Showup 2) as predictors. Because target-presence for the first and second showups did not vary in Experiment 1, target-presence for Showups 1 and 2 were included as a single predictor.

In the initial analyses for Showup 2, we included all main effects in the equation. In the initial analyses for Showup 3, we included all main effects and the current target-presence by previous response (selection vs. rejection) interaction. We then sequentially excluded the interaction if non-significant and any non-significant main effects by order of distance from the current decision. However, given our theoretical predictions, previous choosing was always included in the final model. Although we present the results descriptively here, relevant statistics for full models can be found in Table 2 and relevant statistics for final models can be found in Table 3.

**Table 2:** Experiments 1 and 2: Complete models of logistic regressions predicting choosing on showups 2 and 3 based on previous choosing and target-presence.

	*b*	*SE*	Wald	*p*	95% CI for Odds Ratio		
					*Lower*	*Odds*	*Upper*
**Experiment 1 (*N* = 120)** Showup 2							
Choosing 1	-0.10	0.42	0.06	.813	0.40	0.91	2.06
Target-Presence 1 and 2	1.35	0.42	10.41	.001	1.70	3.87	8.79
Constant	-1.17	0.32	13.14	< .001		0.31	
Showup 3							
Choosing 2	-0.28	0.62	0.20	.655	0.22	0.76	2.57
Choosing 1	0.39	0.44	0.78	.376	0.63	1.47	3.45
Target-Presence 3	0.40	0.50	0.62	.432	0.55	1.49	3.98
Target-Presence 1 and 2	-0.56	0.45	1.53	.217	0.24	0.57	1.39
Choosing 2 × TP 3	2.22	0.89	6.12	.013	1.60	9.17	52.54
Constant	-0.78	0.42	3.53	.060		0.46	
**Experiment 2 (*N* = 248)** Showup 2							
Choosing 1	-0.58	0.28	4.22	.040	0.32	0.56	0.97
Target-Presence 2	0.87	0.26	11.24	.001	1.44	2.40	3.99
Target-Presence 1	0.12	0.27	0.21	.650	0.67	1.13	1.91
Constant	-0.97	0.24	16.54	< .001		0.38	
Showup 3							
Choosing 2	0.50	0.40	1.53	.216	0.75	1.64	3.61
Choosing 1	-0.11	0.28	0.17	.679	0.52	0.89	1.53
Target-Presence 3	1.33	0.32	17.34	< .001	2.02	3.78	7.07
Target-Presence 2	-0.10	0.26	0.00	.969	0.60	0.99	1.65
Target-Presence 1	-0.19	0.27	0.50	.481	0.49	0.83	1.40
Choosing 2 × TP 3	-0.15	0.53	0.82	.774	0.30	0.86	2.44
Constant	-1.00	0.29	11.48	.001		0.37	

Variables were coded as follows. Choosing: non-choosing = 0, choosing = 1; target-presence: TA = 0, TP = 1. Experiment 1. Showup 2: *R*^*2*^ = .09 (Cox & Snell), .13 (Nagelkerke). Model χ ^2^(2) = 11.71, *p* = .003; Showup 3: *R*^*2*^ = .15 (Cox & Snell), .19 (Nagelkerke). Model χ ^2^(5) = 19.06, *p* = .002. Experiment 2, N = 248. Showup 2: *R*^*2*^ = .05 (Cox & Snell), .07 (Nagelkerke). Model χ ^2^(3) = 15.30, *p* = .002; Showup 3: *R*^*2*^ = .10 (Cox & Snell), .14 (Nagelkerke). Model χ^2^(6) = 30.67, *p* < .001. CI = Confidence Interval.

**Table 3:** Experiments 1 and 2: Final models of logistic regressions predicting choosing on showups 2 and 3 based on previous choosing and target-presence.

	*b*	*SE*	Wald	*p*	95% CI for Odds Ratio		
					*Lower*	*Odds*	*Upper*
**Experiment 1** Showup 2							
Choosing 1	-0.10	0.42	0.06	.813	0.40	0.91	2.06
Target-Presence 1 and 2	1.35	0.42	10.41	.001	1.70	3.87	8.79
Constant	-1.17	0.32	13.14	< .001		0.31	
Showup 3							
Choosing 2	-0.45	0.60	0.57	.451	0.20	0.64	2.07
Choosing 1	0.23	0.42	0.32	.573	0.56	1.26	2.85
Target-Presence 3	0.36	0.50	0.53	.469	0.54	1.44	3.81
Choosing 2 × TP 3	2.23	0.89	6.31	.012	1.63	9.27	52.62
Constant	-0.91	0.40	5.08	.024		0.40	
Showup 3, reversed^a^							
Choosing 2	1.77	0.65	7.47	.006	1.65	5.88	20.96
Choosing 1	0.23	0.42	0.32	.573	0.56	1.26	2.85
Target-Presence, reversed	-0.36	0.50	0.53	.469	0.26	0.70	1.85
Choosing 2 × TP 3	-2.23	0.89	6.31	.012	0.02	0.11	0.61
Constant	-0.55	0.37	3.21	.137		0.58	
**Experiment 2** Showup 2							
Choosing 1	-0.54	0.27	4.04	.044	0.34	0.58	0.99
Target-Presence 2	0.87	0.26	11.22	.001	1.44	2.39	3.40
Constant	-0.92	0.21	18.70	< .001		0.40	
Showup 3							
Choosing 2	0.40	0.27	2.27	.132	0.89	1.50	2.52
Choosing 1	-0.17	0.26	0.43	.512	0.50	0.84	1.41
Target-Presence 3	1.28	0.26	24.90	< .001	2.17	3.59	5.93
Constant	-1.04	0.23	20.22	< .001		0.35	

Variables were coded as follows. Choosing: non-choosing = 0, choosing = 1; target-presence: TA = 0, TP = 1. Experiment 1. Showup 2: *R*^*2*^ = .09 (Cox & Snell), .13 (Nagelkerke). Model χ ^2^(2) = 11.71, *p* = .003; Showup 3: *R*^*2*^ = .14 (Cox & Snell), 18 (Nagelkerke). Model χ ^2^(3) = 17.50, *p* = .002. In order to examine the target-presence by previous choosing interaction, the variable TP 3 was reverse-coded so that TA = 1, TP = 0.

^a^Showup 3, reversed represents the regression that was conducted using the reverse-coded target-presence variable and reported in results. Experiment 2. Showup 2: *R*^*2*^ = .05 (Cox & Snell), .07 (Nagelkerke). Model χ ^2^(2) = 15.10, *p* = .001; Showup 3: *R*^*2*^ = .10 (Cox & Snell), 14 (Nagelkerke). Model χ^2^(3) = 30.07, *p* < .001.

**Choosing behavior on the second showup.** Only target-presence was a significant predictor in the final model. Participants were more likely to choose when the target was present. However, due to the fact that target-presence for Showups 1 and 2 did not vary within subjects, it is unclear if it is current target-presence, previous target-presence, or both that are associated with choosing behavior for Showup 2.

**Choosing behavior on the third showup.** The current target-presence by previous choosing interaction was significant. Simple effects were examined by reverse-coding target-presence [37]. Results revealed that only when the current trial was target-present, choosing on Showup 2 predicted choosing on Showup 3: the odds of choosing on the third target-present showup were 5.88 times more likely for those who chose on the second showup compared with those who rejected the second showup. In other words, 79% of those who chose on Showup 2 also chose on a target-present Showup 3, while only 39% of those who rejected Showup 2 subsequently chose on a target-present Showup 3.

#### Experiment 2: Testing for sequential dependencies

Analyses for Experiment 2 were analogous to Experiment 1 with the exception that all initial models included the current target-presence by previous response interaction. The analyses presented here include data from all eight target-presence conditions. We additionally re-ran analyses in Experiment 2 using only the data from the four target-presence conditions in Experiment 1 (TA/TA/TA; TA/TA/TP; TP/TP/TP; TP/TP/TA). Isolating these four conditions did not significantly change the results, and we therefore report only the fully-randomised results. Interested persons can the data available on the OSF. See Table 2 for the relevant statistics for full models including all predictors, and Table 3 for the relevant statistics for final models.

**Choosing behavior on the second showup.** As expected, choosing on Showup 1 was a significant predictor of choosing on Showup 2. However, current choosing contrasted previous choosing, so that if participants chose on the first showup, the odds of choosing were 1.72 times *less* likely than the odds of not choosing. In other words, 72% who chose on Showup 1 subsequently rejected Showup 2. Meanwhile 62% of participants who rejected Showup 1 went on to reject Showup 2. The lack of significant interaction for current target-presence by previous choosing indicates that this sequential dependency was not affected by the current presence of the target. However, current target-presence was also a significant predictor for choosing.

**Choosing behavior on the third showup.** For choosing on Showup 3, only current target-presence was a significant predictor.

### Discussion

Experiments 1 and 2 were initial tests for sequential dependencies across multiple showup identification decisions in the context of multiple perpetrator crimes. We expected previous responses (choosing) and previous target-presence to be related to current decisions. While we did find some evidence for sequential dependencies in both experiments, effects were not consistently predictable. Namely, in Experiment 1, we could only predict choosing behavior between the second and third showups if the third showup was target-present and in Experiment 2 we could only predict choosing behavior between the first and second showups. In Experiment 1, when the current trial was target-present, participants who chose on the second showup were more likely to also choose on the third showup compared with those who had rejected the second showup (assimilation). Although we did expect to find an interaction between current target-presence and previous choosing, the interaction operated counter to expectations. In Experiment 2, regardless of target-presence, participants who chose on the first showup, were more likely to not choose on the second showup (contrast). Taken together, results from both Experiments 1 and 2 provide inconsistent evidence for the capacity to predict choosing behavior from previous choosing. This inconsistency is surprising given the theoretical overlap to fields that have robustly produced sequential dependencies, including perception, absolute identification, and, most pertinently, recognition.

In recognition tests, Malmberg and Annis [27] found sequential dependencies between previous and current responses: A hit on a previous trial increased the probability of a hit on a current trial, but previous hits and false alarms also increased the probability of false alarms on a current trial. In essence, participants were more likely to choose on a current trial if they had chosen on a previous one. This effect was replicated with a variety of paired stimuli (e.g., landscape photo pairs, non-word pairs), as well as with a single-item classic recognition test. While the current research retains similarities to these basic recognition paradigms, as well as other contexts in which sequential dependencies have robustly appeared (i.e., perception, categorization tasks [22, 23]), the eyewitness paradigm also presents differences that may explain the inconsistent results reported here.

Consequently, we considered potentially important differences that may explain the inconsistent results reported here. First, the number of stimuli in our experiment differs greatly from a basic recognition paradigm. In a typical recognition experiment, participants are presented with long lists of words or images, given little time to study these items, and are then tested on those items along with never-before-seen items. Conversely, our experiment only included three perpetrators to study over the course of a 2.5 min mock crime video. Although we cannot ignore the possibility that there are simply not enough stimuli being studied, and therefore participants are not uncertain enough to rely on previous responses, the maximum average participant accuracy rates of 65% do suggest that our filler task allowed for sufficient memory decay to induce uncertainty. Meanwhile, sequential dependencies in recognition are thought to be a result of interference from previous trials that affect mnemonic processing during testing. Therefore, it seems more likely that our results reveal a difference during testing rather than a difference during encoding.

A second difference lies in the number of trials during the testing phases. While recognition experiments may have tens or hundreds of test trials, our participants encountered only three. Perhaps this is not a sufficient number of trials for sequential dependencies to arise. Sequential dependencies have been explained through accumulator models, which predict shifts over time based on criterion placement or accumulation starting points (e.g., Selective Attention, Mapping and Ballistic Accumulation; SAMBA; [24, 25]. The SAMBA model, for example, posits that a participant classifying the loudness of a sound (i.e., *soft* vs. *loud*) uses the sound on initial trials to generate a range between which the subsequent sounds are expected to fall. This range establishes how soft the participants can expect a *soft* sound to be and how loud they can expect a *loud* sound to be. When confronted with the task of classifying the sound on the current trial, the observer will compare the sound to the upper and lower range in relation to the loudness of the previous response. Their response will depend upon the strength of the evidence for each of these answers. When a *soft* response is given on the current trial, it is hypothesized that this biases the perception of the sound on the subsequent trial by temporarily reducing the strength of evidence needed to favor another *soft* response. Thus, assimilation arises from the decision making: because the *soft* response now has the advantage, the following trial is more likely to reach the threshold to be classified as *soft*. Contrast, however, arises from the perceptual mechanisms: Because observers are comparing the current sound to the previous one, any change louder or softer can lead to over- and underestimation of the strength of that sound. In this model, assimilation and contrast both occur because the stronger effect (assimilation) eventually decays to give way to the weaker one (contrast [24]). It is possible that such models require an adjustment period over multiple trials in order to calibrate the upper and lower range of perceptual (and in the case of recognition, mnemonic) processing. As a result, the small number of trials present in our experiment might be insufficient for sequential dependencies to arise.

To address the issues outlined above, Experiment 3 used the recognition paradigm in an attempt to replicate and extend the work of Malmberg and Annis ([27] near-pairs condition) using three different categories of stimuli: photos of faces, photos of landscapes (places) and words. Although we did not expect recognition of faces to explain the lack of predictable sequential effects in the above experiments, it is important to note that sequential dependencies have not yet been tested using face recognition stimuli. For the sake of completeness, we compared the new condition of face stimuli to two conditions with stimuli for which sequential dependencies have been detected during recognition tasks (i.e., places and words).

Accordingly, these concerns were translated into three goals: (1) to extend previous research by testing for sequential dependencies on overall responding in face image recognition, (2) if found, to determine if these sequential dependencies translate to predictable choosing behavior, and (3) to examine whether the strength of these effects vary across the testing phase.

We predicted sequential effects would arise across all three sets of stimuli. If sequential dependencies were observed for responses overall, we predicted that sequential effects would be stronger in the second half compared to the first half of testing blocks and thus also expect to be able to predict choosing behavior in late, but not early, test trials.

## Experiment 3

### Ethics statement

This study was approved by the ethical review board of the Faculty of Psychology and Neuroscience of Maastricht University. Participants in Experiment 3 provided consent by clicking the button to continue the experiment.

### Participants and design

One-hundred-fifty participants were recruited from online participation platforms. Five participants were excluded for the following reasons: failing two of the four control questions (1), failing to follow instructions (2), and taking a 20+ min break in the middle of the first testing block (2). Participants with other anomalous data (e.g., low activity during the filler task) were flagged; when exploratory analyses to examine hit rates, false-alarm rates, accuracy, and choosing behavior did not reveal any of these flagged participants to be outliers, their data were retained for all further analyses. The average age of the remaining 145 participants was 22 years (*M* = 22.14, *SD* = 6.49).

Participants were randomly assigned to one of three conditions to study paired stimuli of faces, places, or words. For each study-test block, participants viewed 18 paired target stimuli during the learning phase and were tested on the 36 target stimuli and 36 fillers. Each participant took part in two study-test blocks, therefore each participant studied a total of 72 targets and was tested on a total of 144 total stimuli (72 targets and 72 fillers). Participants were compensated with research participation credit if eligible, or otherwise not compensated.

### Method

#### Materials

**Fig 1:**
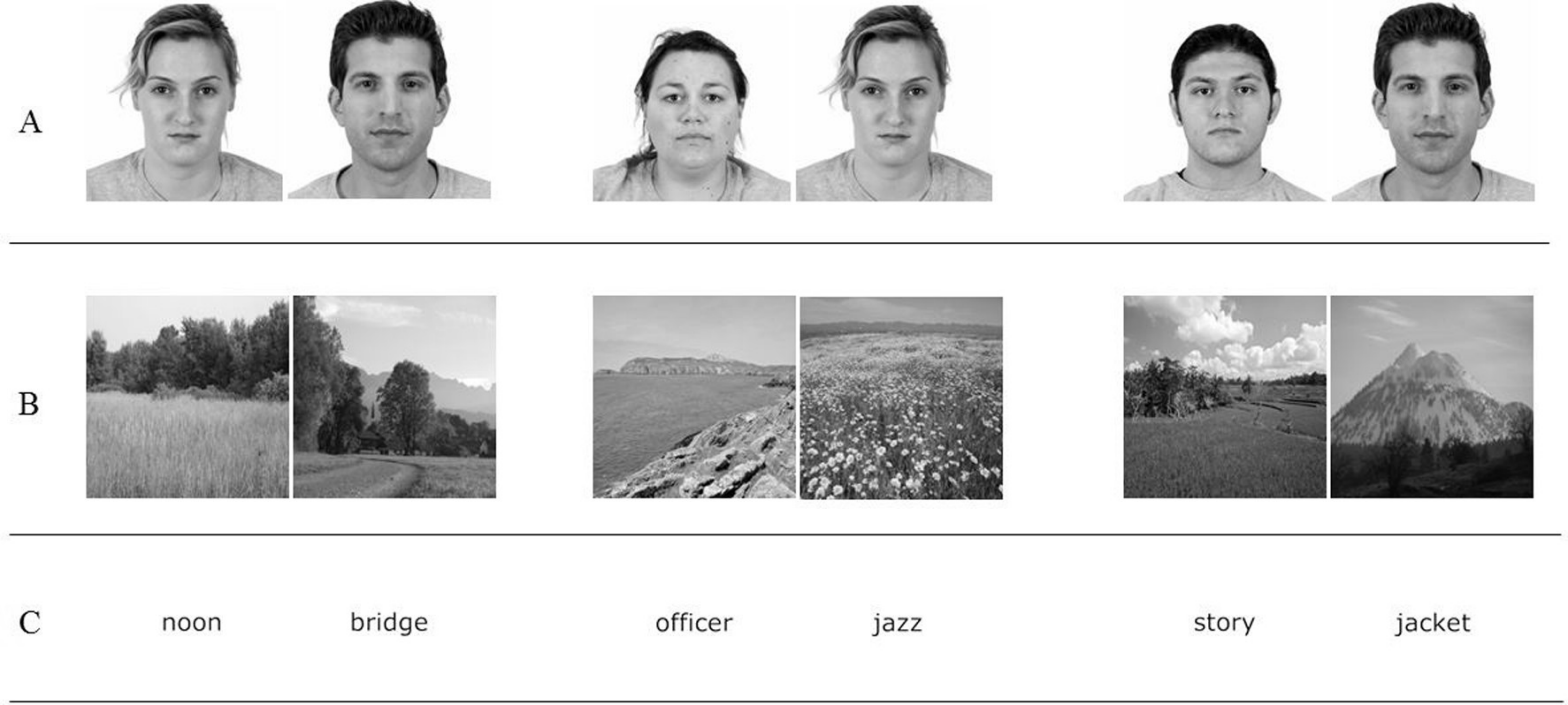
Experiment 3: Example stimuli pairs for faces (Panel A), places (Panel B), and words (Panel C).

See Fig 1 for example stimuli.

**Faces.** Male and female faces with neutral expressions were selected from the Chicago Face Database [38]. Faces that were particularly distinctive, (i.e., shaved eyebrow, facial piercing, unique hair) were removed. Half of the target face stimuli were presented during the learning phase as same-gender pairs and half as opposite-gender pairs. (See Fig 1, Panel A)

**Places.** Photographs of varied landscapes (e.g., sunsets, mountains, deserts, fields) were selected from the Places Scene Recognition Database [39]. Photos with particularly distinctive features (e.g., color filter) were not selected. (See Fig 1, Panel B)

**Words.** One thousand nouns were randomly chosen from the 5,000 most frequently used words according to the Corpus of Contemporary American English [40]. Words were piloted for recognition by seven non-native English speakers whose nationalities are representative of the student population from which the sample is drawn (two Germans, two Belgians, and three Dutch). These non-native English speakers were asked to view the list of 890 nouns and remove those words that they did not recognize (i.e., would need to search for or translate). The stimulus pairs were randomly selected and paired from the remaining 813 nouns. (See Fig 1, Panel C)

#### Procedure

The procedure followed the procedure of Malmberg and Annis ([27] Experiment 1, near-pairs condition replications), with two exceptions. First, because the study was distributed online, a shape appeared at the end of each encoding block (Block 1: blue star, Block 2: black arrow), which was later used as a control question for attention. Second, due to availability of faces, participants studied only 36 total pairs (vs. 40 pairs in the original experiment) of the varying stimuli (faces, places, or words).

Participants were provided a link for the computer task. Participants in the face condition, for example, studied 18 pairs of faces (i.e., 36 faces total). Each pair was presented on screen for 2 s with a 0.1 s interstimulus interval. Following a 30 s distractor task (Pac-Man), participants were presented with two control questions asking them to indicate the form and color of the shape presented at the end of the encoding phase. Participants were then tested for their recognition of the previously-studied faces using the self-paced computer task. Participants in all conditions saw 36 target trials and 36 filler trials of never-before-seen stimuli presented at random, with the constraint that half of the pairs were tested consecutively and the other half were randomized into positions at least seven trials away from their corresponding target trials. Following another 1 min distractor task, this procedure was repeated for a second study-test block. At the end of the experiment, a final question prompted participants to describe the environment in which they completed the experiment (i.e., time of day, location, presence of others).

### Results

We focused on two types of analyses to address the three goals of the experiment. First, we conducted within-subjects tests on overall response patterns to replicate and extend those analyses conducted by Malmberg and Annis [27]. Accordingly, we conducted mixed Analyses of Variance (ANOVAs) on conditional hit rates, false-alarm rates, and choosing rates given previous responses and stimulus type (faces, places, words). Because we were interested in how this effect might vary across the testing sessions, we conducted these same analyses on the conditional hit rates and false-alarm rates for the first half and second half of each of the two testing blocks. We refer to the first half of Block 1 as Section1, the second half as Section 2, and the first and second halves of Block 2 as Sections 3 and 4, respectively.

Second, we conducted between-subjects analyses in order to determine whether overall patterns would be reflected in individual choosing behavior. More specifically, we conducted logistic regressions analogous to those conducted in Experiments 1 and 2 to test whether we could predict choosing behavior on individual trials using target-presence and previous choosing as predictors. Although we present the results descriptively here, relevant statistics for within-subjects analyses can be found in Tables 4 and 5. Statistics for between-subjects analyses can be found in Table 6.

**Table 4:** Experiment 3: Results for ANOVAs and follow-up tests on current hit rates, false-alarm rates, and choosing rates given previous responses and stimulus type.

	*df*	*F*	η^2^	*t*	*d*	*p*
**ANOVAs: Faces, Places, Words**						
Hit Rate: Hit, Miss, FA, CR						
Previous Response	2.25, 321.58	42.57	.229			< .001
Stimulus Type	2, 143	9.00	.112			< .001
Interaction	4.50, 321.58	0.70	.010			.611
FA Rate: Hit, Miss, FA, CR						
Previous Response	2.47, 355.40	26.59	.156			< .001
Stimulus Type	2, 144	9.67	.118			< .001
Interaction	4.94, 355.40	0.67	.009			.646
Choosing: Choose vs. Not						
Previous Choose	1	209.58	.593			< .001
Stimulus Type	2	10.26	.125			< .001
Interaction	2	7.42	.093			.001
Error (within Groups)	144					
**Follow-up t-test: Choosing**						
Faces	45			6.64	1.02	< .001
Places	49			9.76	1.39	< .001
Words	50			9.13	1.30	< .001

The top panel displays results for mixed ANOVAs on hit rates, false-alarm rates, and choosing rates with previous response as the within-subjects factor and stimulus type (faces, places, and words) as the between-subjects condition. False alarm and correct rejection are abbreviated here as FA and CR, respectively. The bottom panel examines the interaction between stimulus type and choosing rates using paired sample t-tests. Although sequential dependencies of choosing appeared in all three stimuli types, the effect was greatest for places, followed by words, and then faces.

**Table 5:** Experiment 3: Results for ANOVAs on current hit rates and false-alarm rates given previous responses and testing section.

	*df*	*F*	η^2^	*p*
**Test sections**^a^**: 1, 2, 3, and 4**				
Hit Rate Contingencies				
Previous Response	3, 318	41.22	. 280	< .001
Test Section	2.44, 259.02	8.63	.075	< .001
Interaction	7.26, 770.03	0.47	.004	.860
False-Alarm Rate Contingencies				
Previous Response	2.73, 305.63	4.85	.042	.004
Test Section	2.72, 304.12	9.06	.075	< .001
Interaction	7.77, 870	0.98	.009	.447

The top panel displays results for repeated-measures ANOVAs on hit rates, false-alarm rates, and choosing rates with previous response (hit, miss, false alarm, correct rejection) and test section (1, 2, 3, 4) as the between-subjects factors.

^a^Sections are broken down into: the first half of the first study-test block (Section 1), the second half of the first block (Section 2), and the first and second halves of the second block (Sections 3 and 4).

**Table 6:** Experiment 3: Results of logistic regression predicting choosing on second and third recognition test trials based on previous choosing and target-presence.

	*b*	*SE*	Wald	*p*	95% CI for Odds Ratio		
					*Lower*	*Odds*	*Upper*
Section 1, trial 3							
Choosing 1	0.57	0.37	2.37	.124	0.86	1.76	3.62
Choosing 2	0.41	0.36	1.28	.258	0.74	1.50	3.03
TP 3	1.62	0.38	18.13	< .001	2.39	5.03	10.58
Constant	-0.97	0.36	7.41	.006		0.38	
Section 2, trial 73							
Choosing 71	0.18	0.38	0.22	.640	0.57	1.20	2.52
Choosing 72	0.79	0.38	4.45	.035	1.06	2.21	4.62
TP 73	0.98	0.38	6.68	.010	1.27	2.67	5.61
Constant	-1.58	0.38	17.04	< .001		0.21	
Section 3, trial 3							
Choosing 1	0.42	0.38	1.22	.269	0.72	1.52	3.20
Choosing 2	-0.32	0.40	0.63	.428	0.33	0.73	1.60
TP 3	-0.70	0.36	3.69	.055	0.24	0.50	1.01
Constant	0.98	0.45	4.71	.030		2.67	
Section 4, trial 73							
Choosing 71	-0.55	0.38	2.12	.145	0.28	0.58	1.21
Choosing 72	0.48	0.37	1.70	.193	0.79	1.61	3.31
TP 73	-0.66	0.36	3.43	.064	0.26	0.52	1.04
Constant	0.83	0.39	4.54	.033		2.30	

Variables were coded as follows. Choosing: non-choosing = 0, choosing = 1; target-presence: TA = 0, TP = 1. Section 1, Trial 3: *R*^*2*^ = .14 (Cox & Snell) .19 (Nagelkerke). Model χ ^2^(3) = 22.50, *p* < .001; Section 2, Trial 73: *R*^*2*^ = .07 (Cox & Snell) .10 (Nagelkerke). Model χ ^2^(3) = 11.33, *p* = .010. Section 3, Trial 3: *R*^*2*^ = .03 (Cox & Snell) .05 (Nagelkerke). Model χ ^2^(3) = 4.98, *p* = .173; Section 4, Trial 73: *R*^*2*^ = .05 (Cox & Snell) .07 (Nagelkerke). Model χ ^2^(3) = 11.33, *p* = .050. CI = Confidence Interval

#### Preparation of data and calculation of contingency rates

Prior to calculating hit rates, false-alarm rates, and choosing rates, trials with response times faster than 200 ms were removed. This is because 200 ms is the approximate threshold for recorded brain activity in response to human faces, as well as the earliest threshold for our ability to distinguish between familiar and unfamiliar faces [41, 42]. Hit rates were calculated as the proportion of correct answers on target-present trials and false-alarm rates were calculated as the proportion of incorrect answers on target-absent trials. Analyses used hit rates on the current trial (*i*) given that the previous trial (*i*-1) was a hit, miss, false alarm or correct rejection. Therefore, separate hit rate contingencies were computed for each participant for (a) hits that followed a hit, HR_hit_ = (H | *i*-1 = hit), (b) hits that followed a miss, HR_miss_ = (HR | *i*-1 = miss), (c) hits that followed a false alarm and, (d) hits that followed a correct rejection. Analogous false-alarm rates for each participant were computed given that the previous response was a hit, miss, false alarm, or correct rejection [27]. Choosing rates were calculated as overall proportion of choosing (respond *yes* vs. *no*) on target-present and target-absent trials.

#### Sequential effects as a function of stimulus type

**Fig 2:**
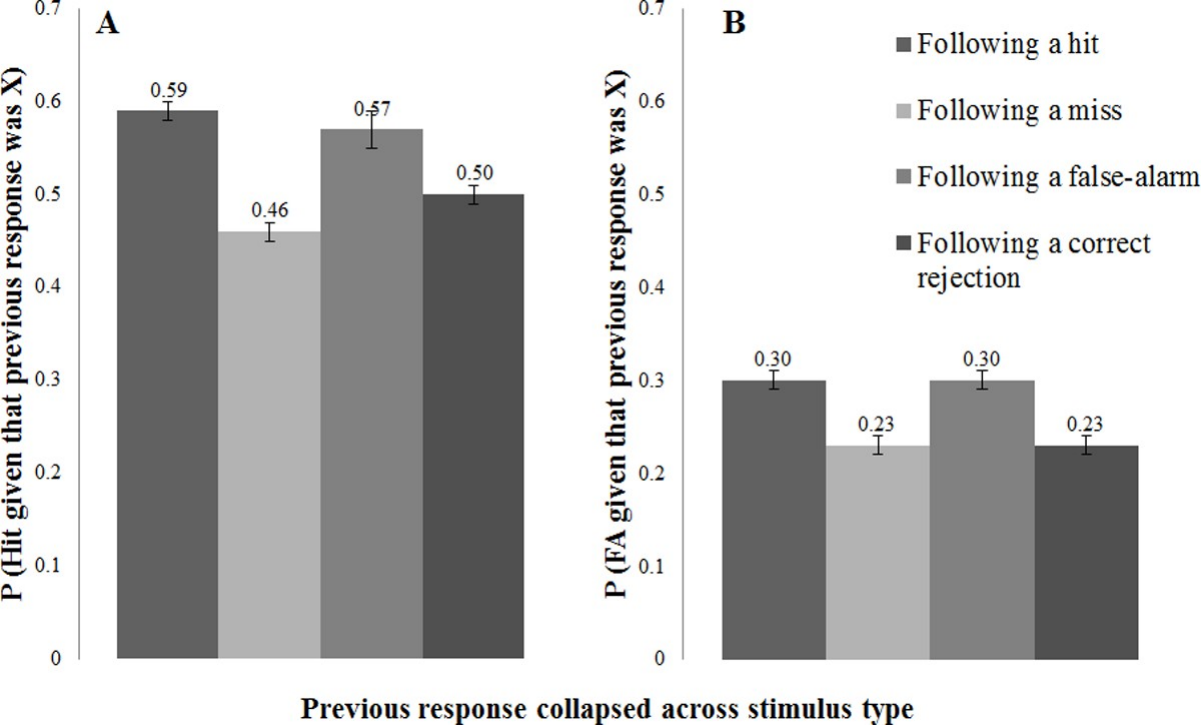
Experiment 3: Hit rate and false-alarm rate contingencies. Panel A displays the probability of a hit on the current trial given the previous response (hit, miss, false alarm, or correct rejection), collapsed across stimulus type (faces, places, words). Previous responses of hit and false alarm do not significantly differ from each other, but all other comparisons are significant (*ps* < .001). Panel B displays the probability of a false alarm on the current trial given the previous response, collapse across stimulus type. A false alarm on the on the current trial is significantly more likely given a previous hit or false alarm when compared with a previous miss or correct rejections (*ps* < .001). Error bars are with standard error.

**Hit rate contingencies.** We examined whether a hit on the current trial (*i*) was more or less likely given a hit, miss, false alarm, or correct rejection on the previous trial (*i*-1), and whether this relationship differed for our three types of stimuli: faces, places, and words. Thus we conducted a mixed ANOVA with previous response being the within-subjects factor, and type of stimulus being the between-subjects factor. There was a significant main effect of previous response. Planned contrasts indicated that a hit on the current trial was more likely if there was either a hit or false alarm compared with a correct rejection or miss on the previous trial. A hit on the current trial was also more likely if there was a correct rejection compared with a miss on the previous trial. The interaction of previous trial by type of stimulus was not significant. Thus, while we found sequential effects for hit rates, these effects did not differ significantly based on whether the stimuli were faces, places, or words. See Fig 2 (Panel A).

**False-alarm rate contingencies**. We next examined whether a false alarm on the current trial (*i*) was more or less likely given a hit, miss, false alarm, or correct rejection on the previous trial (*i*-1), and whether this relationship differed for our three sets of stimuli: faces, places, and words. We conducted a mixed ANOVA with previous response being the within-subjects factor, and stimulus type being the between-subjects factor. There was a significant main effect of previous response, indicating that a false alarm on the current trial was more likely following a hit or false alarm (vs. miss or correct rejection) on the previous trial (see Fig 2, Panel B). There was no significant difference between hits vs. false alarms or misses vs. correct rejections. The non-significant interaction of previous trial with stimulus category provided no evidence that these effects differed significantly according to stimulus type.

#### Sequential effects in choosing

In these analyses, we asked a similar question in a different manner: overall, is choosing (saying *yes*) on the current trial, more or less likely if you chose or did not choose (said *no*) on the previous trial? We conducted mixed ANOVAs with previous choosing (choose vs. not choose) as the within-subjects factor and stimulus type as the between-subjects factor. There was a significant main effect of previous choosing, and a significant interaction between previous choosing and stimulus type. Although sequential dependencies arose within all stimulus categories, the effect was greatest for places, followed by words, and then faces. Together, these results indicate that choosing on the current trial was more likely if the participant chose (vs. did not choose) on the previous trial and that this effect was weakest for face stimuli. See supplementary materials for descriptive statistics (S3 Table).

Given that there were no significant differences between stimulus type for detecting sequential dependencies, all further analyses were collapsed across stimulus type.

#### Sequential effects as a function of section

There were no significant interactions for test section (Sections 1, 2, 3, and 4) with previous response for hits, misses, false alarms, or correct rejections. There was a main effect of test section, such that Sections 1 and 3 displayed higher hit rates and lower false-alarm rates than Sections 2 and 4. Section 1 also displayed higher false-alarm rates than Section 3. See supplementary materials for descriptive statistics (S2 Table).

#### Predicting choosing on individual trials

Given that we successfully replicated analyses demonstrating sequential dependencies in overall recognition memory in the current dataset, we subsequently tested whether those effects would translate to predictable behavior on individual trials over the course of the testing sessions. Therefore, we chose the first three trials of each testing block and the three middle trials of each block (Block 1: trials 1–3 and 71–73; Block 2: trials 1–3 and 71–73). These analyses are of particular interest because they apply analyses from Experiments 1 and 2 to a dataset in which sequential dependencies have already been detected. We consider the first three trials of the first block a proxy for the three showup identification decisions in Experiments 1 and 2. We chose to test the middle trials rather than later trials in order to avoid isolating groups of responses likely to display fatigue effects. Analyses were analogous to Experiment 2 with one exception. Given that there was no current target-presence by previous choosing interaction in Experiment 2, this interaction was not included.

Choosing on the previous trial predicted choosing on the current trial for only one of the four analyses, and current trial target-presence predicted choosing in only trials of Sections 1 and 2. Thus, despite finding that, in general, hits and false alarms were more common when participants chose on the previous trials, behavior on previous trials was not a useful predictor of choosing for these sets of individual trials.

### Discussion

Experiment 3 sought to replicate and extend previous work in sequential dependencies in recognition memory [27]. Except for online data collection and the inclusion of additional questions to control for attention, the procedure followed the near-pairs condition in Malmberg and Annis’ [27] Experiment 1. We expected that sequential dependencies would arise for recognition responses for all three types of stimuli, that these effects would be stronger in later portions of testing, and that this would be reflected in the capacity to predict current choosing from previous choosing in later, but not earlier, test trials.

As expected, the probability of a hit in the current trial (*i*) was higher if the previous response (*i–* 1) was also a hit compared with if the previous response was a miss. The probability of a false alarm on the current trial was increased if it was preceded by either a hit or a false alarm (compared with a miss or correct rejection). This pattern of results did not differ across category of stimuli, meaning we replicated Malmberg and Annis’ results using pictures of places and words, and extended those results to include pictures of faces. Taken together, these results demonstrate that the probability of saying *yes* on the current trial increases any time it is preceded by a *yes* on the previous trial, a conclusion reflected in the analyses conducted on choosing behavior.

We also conducted analyses to determine whether the relationship of previous response reported above changed over the course of the testing session. Contrary to predictions, the effect of previous response did not vary as a function of test section. Although accuracy displayed fatigue effects across the sections (higher accuracy in the first half of each testing block compared with the second half), the strength of sequential dependencies remained constant throughout. Essentially, sequential dependencies did not change across the length of testing.

Lastly, we tested whether these overall effects of sequential dependencies would translate into predictable behavioral outcomes on specific trials. We found little support for the idea that choosing on a previous trial predicted choosing on the current trial. Rather, while sequential dependencies did arise in overall choosing behavior across the total 288 trials, and even the 72 trials comprising each half of the testing blocks, these effects did not reliably arise as predictable choosing behavior on individual trials.

## General discussion

This line of research aimed to answer this key question: in making a series of ostensibly independent showup identification decisions for different perpetrators, is the current decision of an eyewitness related to the previous one(s)? In Experiments 1 and 2, we addressed this question within the eyewitness identification paradigm. Participants watched a mock crime video with three perpetrators and were subsequently asked to make three showup identification decisions, one suspect for each of the perpetrators. Although we found some evidence for sequential dependencies in both experiments, the effect overall was not consistently predictable in regression models. These unexpected results led us to question whether methodological differences between the recognition and eyewitness paradigm could explain the inconsistencies. In particular, we considered whether the number of trials tested influenced the ability to identify these dependencies. Thus, Experiment 3 replicated and extended Malmberg and Annis’ [27] research for sequential dependencies in recognition decisions to test whether (1) sequential dependencies would also arise for face image recognition, (2) these effects could predict choosing behavior on individual trials, and (3) the strength of the above effects varied across the testing session. This approach allowed us to conduct both within-subjects testing to replicate previously reported effects in recognition memory, and the between-subjects modeling applied in Experiments 1 and 2. Experiment 3 showed that sequential dependencies do arise overall for face recognition decisions, that the strength of these effects remains consistent across the testing session, but that these effects do not reliably predict choosing behavior for individual trials. These results and their implications for theory and practice are discussed in turn.

### Sequential dependencies arise for face image recognition decisions

In Experiment 3, we successfully replicated previous research, demonstrating that when participants make a series of yes/no recognition decisions, their responses are affected by the previous trial. A hit on the current trial was more likely when a hit or false alarm (vs. miss or correct rejection) on the previous trial occurred, and a false alarm on the current trial was more likely when either a hit or false alarm (vs. miss or correct rejection) occurred on the previous trial. To confirm this, analyses on choosing behavior established that choosing begets choosing: if participants said *yes* (vs. *no*) on the previous trial, the probability of saying *yes* on the current trial is increased. These effects were found for three types of stimuli, including images of faces. Indeed, in our analyses with hit rate and false-alarm rate contingencies, while the overall contingency rates varied depending upon the stimulus type, the relationship between previous and current response did not. Thus, this experiment adds to a growing list of decisions in which sequential dependencies arise, including detection of sound [22], ratings of sweetness in wine taste-tests [43], and judgements of frequency in landscape recognition [44].

### Effects are consistent across testing

Next, we tested whether the strength of sequential dependencies varied across the length of the testing session. Accumulator models used to explain sequential dependencies predict shifts over time based on variation in criterion placement or accumulation starting points (e.g., SAMBA [24, 25]). We hypothesized that effects would be stronger in the second half of each testing session compared with the first half of each session. Indeed, Schifferstein and Kuiper [45] removed the first 20 “outlier” responses of their experiment tasting aqueous solutions because high response variability is greatest in these initial trials. Contrary to expectations, the strength of sequential dependencies remained constant across the length of the testing session.

In sum, our results established that sequential dependencies arise consistently within participants separately from individual differences in criteria. We could therefore be certain that our results replicated previous experiments on sequential dependencies as we transitioned to apply the regression models used in Experiments 1 and 2.

### Sequential dependencies are not reflected as predictable choosing behavior

We next tested whether these dependencies would also predict behavioral outcomes on the first three and middle three trials of each testing block. The first three trials of the first block are of greatest interest because they best represent the three showup identification trials in Experiments 1 and 2. Consistent with Experiments 1 and 2, and despite detecting sequential dependencies in overall responses, we were not able to detect sequential dependencies in individual trials. Given that the strength of sequential dependencies detected by within-subjects analyses did not vary across the testing session, it was unsurprising to find that detecting sequential dependencies on individual trials did not differ. Critically, these results appear to be good news for the eyewitness context. We were originally concerned that multiple identification decisions may give rise to sequential dependencies, and thus affect the integrity of the identification decisions being made. However, this is not the case. If we cannot predict current recognition decisions from previous ones, then there is less reason to believe that dependencies are likely to be problematic for the multiple high-stakes recognition decisions in the eyewitness identification context.

These results are in line with a recent study that considers the effect of making multiple lineup decisions, although not within the theoretical framework of sequential dependencies [46]. In that study, participants watched 24 videos, each followed by lineup identification decisions on target-present and target-absent trials. The authors found that the number of trials had either no effect or a trivial effect on accuracy, choosing, or confidence. This is not to say that a series of identification decisions cannot possibly be related to each other. Indeed, sequential dependencies are only one way in which the assumption of independence may be violated between multiple decisions. Research on probability matching [47] demonstrates that people making a series of decisions use a response strategy that reflects their beliefs about base rates for the task. For example, students may avoid circling the choice (*B*) too many times on a multiple-choice test because they believe that correct answers are likely to be evenly distributed across the listed options. It is possible that eyewitnesses confronted with multiple lineups are influenced by these expectations of base rates (i.e., the police probably detained all three perpetrators vs. the experimenter would never show me all three perpetrators). In other words, though we have ruled out one possibility on the relationship between multiple identification decisions, there is more to be investigated.

### Conclusion

Neither the use of faces nor differences in the number of trials could explain the contradictory results in Experiments 1 and 2 that we sought to resolve. However, the inability to use previous choosing behavior as a predictor for current choosing in Experiment 3, a data set that we know contains sequential dependencies, still serves to clarify our previous contradictions. We suspect that the discrepancy between detecting sequential dependencies in overall responses using within-subjects analyses and not on individual responses with regression models is an indication of weak effects. The within-subjects ANOVAs provide the statistical power to detect small effects, while the regression models do not. In this case, probabilities of choosing on current trials are heightened by previous choosing over many opportunities to choose or not to choose, but these effects do not translate to detectable behavioral outcomes of choosing on individual trials. In each of three experiments, it was sometimes possible to predict current choosing from previous choosing, but not reliably so, and often not in the expected direction.

In summary, sequential dependencies arise in face image recognition, and though the accuracy across stimuli and section of testing session may vary, the pattern of dependencies does not change. However, these effects do not translate to individual trials, and we therefore suggest that the integrity of identification and recognition decisions is not likely to be impacted by making the multiple decisions in a row. This is the first paper to systematically explore sequential dependencies in face recognition and particularly in eyewitness identification, contributing to the small, but vital, literature that aims to disentangle factors underlying the decreased performance in recognition for multiple faces. It thus contributes towards the eventual goal to offer procedural recommendations adapted to the difficulties present in the administration of identification procedures in the context of multiple perpetrator crimes.

## Supporting information

S1 Table Pilot study Experiments 1 and 2: Mean (standard deviation) age, distinctiveness, memorability, typicality and similarity values for target faces and corresponding innocent suspect. *Note*: Participants were shown each of the photographs (targets and replacements) individually and were asked to estimate age and to rate distinctiveness and memorability on a five-point scale from 1 (*not at all distinctive/memorable*) to 5 (*extremely distinctive/memorable*) and to rate deviation from typicality (*How much would this face have to be modified to look completely typical/average*?) on a scale from 0 (*no modification*) to 5. Participants indicated how similar they considered the two faces on a scale from 1 (*not at all similar*) to 5 (*very similar*). Innocent suspects were rated as statistically non-different to the perpetrator for the following three factors: memorability, distinctiveness, and deviation from typicality. Innocent suspects 2 and 3 significantly differed in age from their respective perpetrators: Suspect 2: *t*(21) = 2.73, *p* = .013; Suspect 3: *t*(21) = -6.41, *p* ≥ .001. Perpetrators and their corresponding innocent suspects were also rated for similarity. These tests revealed no significant differences between pairs; *p*s ≥ .162.
(DOCX)

S2 Table Experiment 3: Hit rates and false-alarm rates (standard error) given previous response as a function of test section. *Note*. Participants took part in two study-test blocks. Sections represent the first half of the first block (Section 1), the second half of the first block (Section 2), and the first and second halves of the second block (Sections 3 and 4).
(DOCX)

S3 Table Experiment 3: Choosing rates (standard error) given previous choosing as a function of stimulus type. (DOCX)
