# Supplementary material for: Showup identification decisions for multiple perpetrator crimes: Testing for sequential dependencies
Source: PLoS One. 2018 Dec 6;13(12):e0208403. doi: 10.1371/journal.pone.0208403 (PMC6283529; doi:10.1371/journal.pone.0208403)
Supplement: S1 Table — Note: Participants were shown each of the photographs (targets and replacements) individually and were asked to estimate age and to rate distinctiveness and memorability on a five-point scale from 1 (not at all distinctive/memorable) to 5 (extremely distinctive/memorable) and to rate deviation from typicality (How much would this face have to be modified to look completely typical/average?) on a scale from 0 (no modification) to 5. Participants indicated how similar they considered the two faces on a scale from 1 (not at all similar) to 5 (very similar). Innocent suspects were rated as statistically non-different to the perpetrator for the following three factors: memorability, distinctiveness, and deviation from typicality. Innocent suspects 2 and 3 significantly differed in age from their respective perpetrators: Suspect 2: t(21) = 2.73, p = .013; Suspect 3: t(21) = -6.41, p ≥ .001. Perpetrators and their corresponding innocent suspects were also rated for similarity. These tests revealed no significant differences between pairs; ps ≥ .162. (DOCX) [file pone.0208403.s001.docx]

**S1 Table. Pilot Study: Mean (standard deviation) Age, Distinctiveness, Memorability, Typicality and Similarity Values for Target Faces and Corresponding Innocent Suspect**

|  | Perpetrator 1  *M* (*SD*) | Suspect 1  *M* (*SD*) | Perpetrator 2  *M* (*SD*) | Suspect 2  *M* (*SD*) | Perpetrator 3  *M* (*SD*) | Suspect 3  *M* (*SD*) |
| --- | --- | --- | --- | --- | --- | --- |
| Age | 23.45 (1.47) | 22.91 (2.76) | 22 (2.05) | 23.27 (2.76) | 25.14 (1.64) | 22.73 (2.41) |
| Distinctiveness | 2.95 (1.00) | 2.82 (1.22) | 3.14 (0.94) | 2.86 (0.99) | 3.77 (0.97) | 3.45 (1.06) |
| Memorability | 3.00 (1.07) | 2.59 (1.14) | 2.00 (1.16) | 2.73 (0.83) | 3.50 (1.01) | 3.27 (0.99) |
| Typicality | 1.72 (0.98) | 1.72 (1.12) | 1.86 (1.08) | 1.64 (1.29) | 2.00 (1.02) | 2.05 (1.09) |
| Similarity | 2.81 (0.80) | | 2.45 (0.96) | | 2.45 (0.91) | |

*Note*: Participants were shown each of the photographs (targets and replacements) individually and were asked to estimate age and to rate distinctiveness and memorability on a five-point scale from 1 (*not at all distinctive/memorable*) to 5 (*extremely distinctive/memorable*) and to rate deviation from typicality (*How much would this face have to be modified to look completely typical/average?*) on a scale from 0 (*no modification*) to 5. Participants indicated how similar they considered the two faces on a scale from 1 (*not at all similar*) to 5 (*very similar*). Innocent suspects were rated as statistically non-different to the perpetrator for the following three factors: memorability, distinctiveness, and deviation from typicality. Innocent suspects 2 and 3 significantly differed in age from their respective perpetrators: Suspect 2: *t*(21) = 2.73, *p* = .013; Suspect 3: *t*(21) = -6.41, *p* < .001. Perpetrators and their corresponding innocent suspects were also rated for similarity. These tests revealed no significant differences between pairs; *p*s ≥ .162.
