# Supplementary material for: Showup identification decisions for multiple perpetrator crimes: Testing for sequential dependencies
Source: PLoS One. 2018 Dec 6;13(12):e0208403. doi: 10.1371/journal.pone.0208403 (PMC6283529; doi:10.1371/journal.pone.0208403)
Supplement: S2 Table — Note. Participants took part in two study-test blocks. Sections represent the first half of the first block (Section 1), the second half of the first block (Section 2), and the first and second halves of the second block (Sections 3 and 4). (DOCX) [file pone.0208403.s002.docx]

**S2 Table. Experiment 3: Hit Rates and False-Alarm Rates (Standard Error) Given Previous Response as a Function of Test Section**

|  | Hit Rate | | | |  | FA Rate | | | |
| --- | --- | --- | --- | --- | --- | --- | --- | --- | --- |
|  | Hit | Miss | FA | CR |  | Hit | Miss | FA | CR Rejection |
| **Section** |  |  |  |  |  |  |  |  |  |
| 1 | .66 (.02) | .59 (.02) | .66 (.02) | .62 (.02) |  | .34 (.02) | .28 (.02) | .31 (.02) | .29 (.01) |
| 2 | .52 (.02) | .47 (.02) | .50 (.03) | .49 (.02) |  | .29 (.02) | .24 (.02) | .27 (.02) | .26 (.02) |
| 3 | .63 (.02) | .56 (.03) | .60 (.03) | .56 (.02) |  | .35 (.02) | .26 (.02) | .31 (.02) | .28 (.02) |
| 4 | .52 (.02) | .45 (.02) | .51 (.03) | .44 (.02) |  | .29 (.02) | .27 (.02) | .29 (.03) | .23 (.02) |

*Note.* Participants took part in two study-test blocks. Sections represent the first half of the first block (Section 1), the second half of the first block (Section 2), and the first and second halves of the second block (Sections 3 and 4).
