# Supplementary material for: Showup identification decisions for multiple perpetrator crimes: Testing for sequential dependencies
Source: PLoS One. 2018 Dec 6;13(12):e0208403. doi: 10.1371/journal.pone.0208403 (PMC6283529; doi:10.1371/journal.pone.0208403)
Supplement: S3 Table — (DOCX) [file pone.0208403.s003.docx]

**S3 Table. Experiment 3: Choosing Rates (Standard Error) Given Previous Choosing as a Function of Stimulus Type**

|  | Choosing Rates | |  |
| --- | --- | --- | --- |
|  | Choose | Not Choose |  |
| **Section** |  |  |  |
| Faces | .58 (.01) | .51 (.01) |  |
| Places | .57 (.01) | .47 (.01) |  |
| Words | .64 (.01) | .50 (.01) |  |
